# Systemic stress test model for shared portfolio networks

**DOI:** 10.1038/s41598-021-82904-y

**Published:** 2021-02-08

**Authors:** Irena Vodenska, Nima Dehmamy, Alexander P. Becker, Sergey V. Buldyrev, Shlomo Havlin

**Affiliations:** 1 Department of Administrative Sciences, Metropolitan College, Boston University, 1010 Commonwealth Avenue, Boston, MA 02215 USA; 2 Center for Polymer Studies and Department of Physics, Boston University, 590 Commonwealth Avenue, Boston, MA 02215 USA; 3 Center for Science of Science and Innovation, Kellogg School of Management, Northwestern University, Evanston, IL 60208 USA; 4 Department of Physics, Yeshiva University, 500 West 185th Street, New York, NY 10033 USA; 5 Bar-Ilan University, 52900 Ramat Gan, Israel

**Keywords:** Complex networks, Phase transitions and critical phenomena, Nonlinear phenomena

## Abstract

We propose a dynamic model for systemic risk using a bipartite network of banks and assets in which the weight of links and node attributes vary over time. Using market data and bank asset holdings, we are able to estimate a single parameter as an indicator of the stability of the financial system. We apply the model to the European sovereign debt crisis and observe that the results closely match real-world events (e.g., the high risk of Greek sovereign bonds and the distress of Greek banks). Our model could become complementary to existing stress tests, incorporating the contribution of interconnectivity of the banks to systemic risk in time-dependent networks. Additionally, we propose an institutional systemic importance ranking, *BankRank*, for the financial institutions analyzed in this study to assess the contribution of individual banks to the overall systemic risk.

Recent financial crises have motivated the scientific community to seek new interdisciplinary approaches to modeling the dynamics of global economic systems. Following seminal papers by Allen and Gale^1,2^, a number of empirical studies have attempted to estimate the risk of contagion in financial systems: the UK interbank market^3^, the German interbank market^4^, or the Austrian interbank market^5^, to name a few. Other works^6,7^ turned their attention to the impact of leverage and liquidity risk on contagion. While many of the existing economic models include noise and fluctuations, they assume a representative economic agent or use market data to infer interdependencies among financial institutions. One such approach analyzes the exposure of financial institutions to common macro-factors to find the systemic risk impact that banks have on each other^8,9^. However, this type of analysis generally does not take into account the structure of the underlying economic network. In the aftermath of the global financial crisis, researchers argued for a new approach^10^. Economists have recently expanded traditional econometrics modeling with increased attention to two factors: (i) the structure of economic networks and (ii) their dynamics. One example of this approach is DebtRank^11^. The authors study the 2008 banking crisis and use network analysis to assess the role a bank plays in propagating systemic risk to other banks. By defining the DebtRank, a dynamic centrality measure in the interbank lending network, they show that the banks that need to be rescued are the ones that are more “central” in terms of their DebtRank. Identifying the critical nodes in the network is crucial since recent work has shown that nodes with higher centrality have greater potential for triggering cascading failures^12–16^. DebtRank has also been extended to bipartite networks, e.g., to the lending relationships between banks and firms in Japan^17^, however, the authors did not take in consideration the dynamic behavior of link weights.

Our paper is motivated by the European sovereign debt crisis that began in late 2009 with the divergence of the yield on Greek sovereign debt compared to the yield on debt of other European nations and led to a bailout of the Greek government^18^. The nature of the sovereign debt crisis in 2011 and the resulting network behavior differs from the 2008 global financial crisis. Here we focus on studying investment funds and financial institutions that have been major holders of sovereign debts of Greece, Italy, Ireland, Portugal, and Spain (GIIPS), most affected eurozone countries by the European sovereign debt crisis. When these governments encountered fiscal difficulties, the banks holding their sovereign debt faced a dilemma, either to divest some or all of their holdings at reduced values or to try and wait out the crisis. In contrast to studies that focus on interbank lending^11,19,20^, our work explores the systemic risk impact of portfolio overlap. The similarity of banks’ asset holdings as a channel of contagion has recently attracted increased attention^21–23^.

We propose a dynamic network model for assessing the vulnerability of the financial system to economic shocks. We construct a network of the largest institutional holders of sovereign debt of the five troubled eurozone countries (Greece, Italy, Ireland, Portugal, and Spain) during the European sovereign debt crisis of 2010-2012. We also study the potency of individual banks to propagate systemic risk throughout the bank network. We propose a measure for a bank’s systemic importance in shared portfolio networks, *BankRank*, to identify how the linkages of banks contribute to the overall network losses in bank assets. In simulations we determine whether the network is in a stable state in which shocks do not cause major losses, or it is in an unstable state in which devastating damages occur. While the largest sovereign debt holders are usually more important, in the unstable regime smaller holders also exhibit systemic importance. Measuring the parameters of our model from the eurozone crisis data, the results show that before the crisis, the system was mostly in a stable regime, and that during the crisis it transitioned into an unstable regime. The numerical solutions produced by our model match closely the actual timeline of events of the crisis. Our model thus may be a useful tool for simulating the response dynamics of shared portfolio networks. We stipulate that our model can improve the ability to estimate the vulnerability of banks and assets to shocks. Using a snapshot of the GIIPS sovereign debt holders network from the end of 2011, we observe that:

1. When we model the system’s response to an individual bank experiencing a shock, our analysis is in accordance with real-world results, e.g., in our simulations, Greek debt is clearly the most vulnerable.
2. The dynamics arising from our model produce different outcomes for the system depending on the values of the parameters. The system exhibits at least two equilibria; in one, the system suffers mild monetary damage, while in the other, the monetary damage is quite significant and devastating.

In addition to DebtRank^11^, our paper also builds on recent studies of cascading failures in a bipartite network of banks and assets in which risk propagates among banks through overlapping portfolios^22,24–27^. Other models use simulated networks similar to real systems^28^, allow dynamic behavior of the nodes but not the links^29^, or incorporate dynamic behavior when a financial network attempts to optimize “risk-adjusted” assets^30,31^. Our approach expands upon these earlier models; with only two parameters, and all network variables are dynamic.

## Results

**Figure 1:**
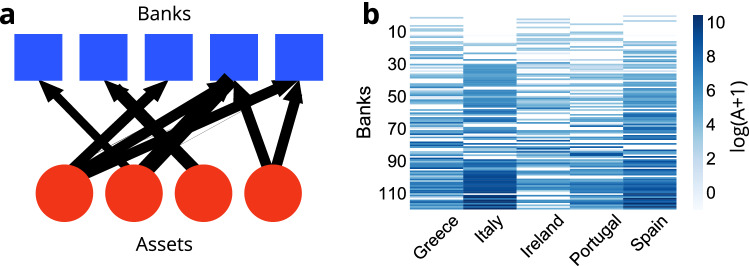
(**a**) A sketch of the network of banks vs assets as a weighted bipartite graph. The thickness of the lines represents sovereign debt holding weights. The network is characterized by its weighted adjacency matrix *A*. The entries $A_{i\mu }$ describe the number of bonds $\mu$ held by bank *i*. (**b**) Amount of banks’ holdings in GIIPS sovereign debt expressed in units of millions of euros. The vertical axis denotes different banks, and they are sorted from top to bottom in ascending order in terms of their total exposures to GIIPS debt. Because holdings differ by orders of magnitude, we have plotted $\log (A+1)$ here.

We develop a dynamic risk propagation model using a bipartite network with banks in one part and assets in the other, as shown in Fig. 1. While our model is general, we are focusing on sovereign debt as the asset that drives the dynamics of the system. Governments borrow money by issuing sovereign bonds that trade in a secondary market, similar to the stock market^32^ where the laws of supply and demand determine the value of the bonds. Typically, investors consider sovereign bonds of developed countries as risk-free or very low risk. If, however, a country becomes troubled and the market perceives that the government may not be able to pay back its debt, the price of the sovereign bonds can crash. During the European sovereign debt crisis, Greek debt exhibited such behavior.

### Model setup

Each bank is endowed with equity and a portfolio of assets. The bank’s holdings determine the weighted links to the asset part of the bipartite network. Both equity and asset holdings may change dynamically over time. Each asset initially trades at its par value, and depending on supply and demand, its price will adjust. Banks are constrained by regulation, influencing their reactions to changes in the value of their holdings, particularly if losses in their portfolio negatively affect their liquid capital. Commensurate with the observed change in equity, after a short delay, banks will buy or sell assets. Our model includes a parameter for bank behavior, a “panic factor,” which diminishes or amplifies the response of banks to asset value changes. Any action by the banks results in a shift in supply and demand and, after a short delay, affects asset prices. The change in asset prices is proportional to the fraction of assets being bought or sold and to the liquidity of the asset. Our model includes a “liquidity” parameter measuring the depth of the market. For example, if a bank sells a small amount of a particular asset or if the asset is very liquid, the price impact will be minimal. If, however, banks sell a large amount of an asset or if the asset is particularly illiquid, the price changes will be magnified. We also make the following additional key assumptions and specifications in our model.

- *Open system* The banks do not exclusively trade with each other. They may trade with an external entity, which may be the central bank or other, smaller investors. With respect to actual financial networks, excluding other actors would be an unrealistic assumption. Take the case of GIIPS sovereign debt; in addition to the European Central Bank (ECB), which has been buying some of the bonds if there was a need to stabilize the system, a large number of investors hold this debt. In this way, our model is distinct because agents are not assumed to be trading only with each other, as is the case in many other models in banking or financial networks.
- *Equilibrium perturbation* When there is no change in equity, price of the sovereign bonds, or bond holdings, there is no intrinsic dynamic activity in our financial network.
- *Herding* Agents in the system (institutional holders of sovereign debt) follow other agents’ actions leading to the so-called “herding effect.”
- *Short-term response* The model describes the short-term responses of the system and disregards slow, long-term driving forces of the market.

One part of the network contains bonds, or assets, ${\mathcal {A}}$, which we label using Greek indices. In the following, we use the terms “bond” and “asset” interchangeably. The other part of the network contains a set of banks ${\mathcal {B}}$, which we label using Roman indices. When a bank owns an asset, a link between the respective bank *i* and asset $\mu$ is formed. The weight of the link indicates the quantity of asset $\mu$ that bank *i* owns. Also, for a bank, holding sovereign debt is equivalent to the bank lending funds to a government. Each bank is characterized by its equity $E_t(t)$ and its holdings from the set of assets $A_{i\mu }(t)$, which both change over time and generally differ from bank to bank. At any given time *t*, bond $\mu$ can be bought or sold for price $P_{\mu }(t)$. This price depends on the supply and demand for this asset. Without loss of generality, all bonds start trading at par, that is, $P_\mu (0)=1$.

Our model allows us to describe how each of the variables $E_i(t), A_{i\mu }(t)$, and $P_{\mu }(t)$ evolves over time. A key feature of our model is the time-dependence of the links $A_{i\mu }$, introducing dynamics into our network. The equity $E_i(t)$ of a bank *i* is

1 $$\begin{aligned} E_i(t)= \max \left\{ \sum _{\mu \in {\mathcal {A}}} A_{i\mu }(t)P_{\mu }(t) + C_i(t)\,, \;0\right\} . \end{aligned}$$

Here $P_{\mu }$ is the price of asset $\mu$ as a fraction of its original price. If the assets are bonds, this corresponds to the fraction of their par value. $C_{i}$ denotes the liquid capital of the bank. These parameters evolve in time. We use the maximum function to denote that a bank’s equity is non-negative; if the equity of bank drops below zero in our model, the bank goes bankrupt. We further introduce the market value of the bond portfolio held by bank *i*, $V_i\equiv \sum _{\mu \in {\mathcal {A}}} A_{i\mu }P_\mu$, and the par amount of bond $\mu$ outstanding, $A_\mu \equiv \sum _{i\in {\mathcal {B}}} A_{i\mu }$. While the liquid capital of the bank is subject to change for a myriad of reasons, we focus on the impact of sales and purchases of assets from set ${\mathcal {A}}$. For that reason, we separate out the change in liquid capital due to portfolio transactions from other sources of change in liquid capital:

2 $$\begin{aligned} \Delta C_i(t)= -\sum _{\mu \in {\mathcal {A}}} (\Delta A_{i\mu }(t)) P_{\mu }(t)+\Delta S_i(t). \end{aligned}$$

The minus sign indicates that a sale of assets implies $\Delta A_{i\mu }<0$, and the transaction should increase the liquid capital on the balance sheet of bank *i*. $\Delta S_i$ encompasses the impact due to other sources of capital changes.

Due to regulatory constraints, the bank is concerned how changes in liquid capital affect its equity. A sale of an asset, as described in Eq. (2), does not decrease the bank’s equity because the reduction in assets corresponds to a commensurate increase in the cash position. $\Delta S_i$, however, describes the net impact of other factors on liquid capital, and therefore, it has an impact on the bank’s equity. A change in the prices of bonds from the set of assets ${\mathcal {A}}$ also changes the bank’s equity:

3 $$\begin{aligned} \Delta E_i(t)= \Delta S_i(t) + \sum _{\mu \in {\mathcal {A}}} A_{i\mu }(t) \Delta P_{\mu }(t). \end{aligned}$$

If a bank’s equity shrinks, it may need to consider selling assets, possibly at depressed prices, while, if the equity increases, the bank may expand its holdings. In our model, the bank decides to buy or sell assets based on the changes in its portfolio (or indirectly, the change in its equity):

4 $$\begin{aligned} \Delta A_{i\mu }(t+\tau _A) = \beta \,\frac{\Delta E_t(t)}{E_t(t)}\; A_{i\mu }(t). \end{aligned}$$

We introduce $\beta$ to model the bank’s urgency to purchase or sell assets. The larger $\beta$ is, the more assets the bank trades as a response to a change in portfolio value. In the case of asset sales, $\beta$ can be regarded as a ‘panic factor’ as it accelerates the selling pressure on the assets. Our model assumes that the bank acts after a response time $\tau_A$ following the change in its equity position. When banks trade, they impact asset prices. In our model, price changes are proportional to the quantity of assets sold as a fraction of total assets held by other institutions in the set of banks ${\mathcal {B}}$ and the market sensitivity to sales or market liquidity. The price impact of asset sales is not immediate in our model; instead, we account for a short market response time $\tau_P$.

5 $$\begin{aligned} \Delta P_{\mu }(t+\tau _P) =\alpha \,\frac{\Delta A_{\mu }(t)}{A_{\mu }(t)}\;P_{\mu }(t), \end{aligned}$$

where $\alpha$ is the market sensitivity. The fraction of sales ($\Delta A/A$) required to reduce the price by one unit ($\Delta P /P$) is equal to $1/\alpha$. Therefore, this parameter can also be understood as the inverse of market liquidity or market depth. We assume the same “inverse market depth” $\alpha$ for all assets in ${\mathcal {A}}$. For notation description, see Table 1.

**Table 1:** Notation.

$A_{i\mu }(t)$	Holdings of bank *i* in asset $\mu$ at time *t*
$P_{\mu }(t)$	Fraction of price of asset $\mu$ at time *t*; $P_{\mu }(0)=1$
$E_t(t)$	Equity of bank *i* at time *t*
$\alpha$	Inverse market depth
$\beta$	Banks’ panic factor
$\tau _A, \tau _P$	Response times of investors and prices, respectively

So far, we have used discrete time steps. Assuming small time lags, we convert Eqs. (3)–(5) to continuous-time differential equations. We transform $\Delta F \rightarrow \mathrm {d}F/\mathrm {d}t$ and expand the resulting equations to second order in time (If the time lags are small, we can expand the equations with $\tau$ to $\frac{\mathrm {d}F(t+\tau )}{\mathrm {d}t}\approx \frac{\mathrm {d}}{\mathrm {d}t}\left( F(t)+\tau \frac{\mathrm {d}F}{\mathrm {d}t}\right) = \frac{\mathrm {d}F}{\mathrm {d}{t}} + \tau \frac{\mathrm {d^2}F}{\mathrm {d}{t^2}}$). Further, we will assume the response times of the market and the banks are identical $\tau = \tau _A=\tau _P$. Differences in response time do not affect the the stability of the system (see Supplementary Section S1). The equations of the model can be written as:

6 $$\begin{aligned} \left( \tau \partial _t^2 +\partial _t \right) A_{i\mu }(t)&=\beta \,\frac{\partial _t E_i(t)}{E_i(t)} \;A_{i\mu }(t) \end{aligned}$$

7 $$\begin{aligned} \left( \tau \partial _t^2 +\partial _t \right) P_\mu (t)&=\alpha \,\frac{\partial _t A_\mu (t)}{A_\mu (t)}\;P_\mu (t) \end{aligned}$$

8 $$\begin{aligned} \partial _t E_i(t)&= f_i(t) + \sum _{\mu \in {\mathcal {A}}} A_{i\mu }(t) \,\partial _t P_\mu (t). \end{aligned}$$

where $f_i(t)=\mathrm {d}S_i/\mathrm {d}t$ is the impact of external influences, and $\tau$ is the time-scale in which banks and markets respond to a change. Without such a time lags, Eqs. (6)–(8) would merely relate the first-order time derivatives of *E*, *A*, *P* to each other, similar to Eq. (11), and there would not be any dynamics.

### Shocking the system

In Eq. (8), $f_i(t)$ denotes changes to the liquid capital and the bank’s equity from other sources than the changes of bank’s portfolio of assets ${\mathcal {A}}$. We assume that a shock to the system comes in the form of $f_i(t) = s E_i \delta (t)$, where $\delta (t)$ is the Dirac delta function. Such a shock instantaneously changes the equity of a bank *i* by a fraction *s* of its equity and leaves all other banks unaffected, $f_j=0$ for $j\ne i$. Inserting $f_i(t)$ into Eq. (6), we can find the initial condition for $A_{i\mu }$ through integration:

9 $$\begin{aligned} \partial _tA_{i\mu }(0)&= \beta A_{i\mu }(0) \ln (1+s). \end{aligned}$$

Since the other banks are initially unaffected by this shock, their holdings are not modified. This implies $\partial _t A_{j\mu }(0)=0$ for $j\ne i$. Additionally, the $f_i(t)$ that we are using implies that the initial equity of bank *i* changes to $(1+s)E_i(0)$. If $s<0$, then the bank experiences a deterioration of its equity. Naturally, asset prices can not be negative, implying $P_{\mu }(t)\ge 0$. Our model does not allow short sales, hence $A_{i\mu }(t)\ge 0$. Lastly, the bank’s equity cannot fall below zero, which means $E_{i}(t)\ge 0$, as described in Eq. (1).

### Linear response model

We are interested in the response of the system to external shocks. We assume that, before being shocked, the system is near equilibrium. This equilibrium may correspond to local optima of an objective function (Lagrangian) ${\mathcal {L}}(E,A,P,S)$, with *S* representing exogenous drivers or shocks. We perform a Taylor expansion of ${\mathcal {L}}$ near an equilibrium point and keep the leading order terms. The possible terms that thus may appear in the expansion of ${\mathcal {L}}$ are of the form $|E|^2, |P|^2, |A|^2$, as well as $E^TAP$ and their various time derivatives. For brevity, we define $\partial _t\equiv \frac{\mathrm {d}}{\mathrm {d}t}$. Although terms like $\partial _t E^TAP$ are third order in the variables, they are the only terms which utilize the network structure to connect losses or gains $\partial _t E$ to asset prices, thereby serving as channels for risk propagation. Hence we have

10 $$\begin{aligned} {\mathcal {L}}(E,A,P) \approx {1\over T}\int \mathrm {d} t \left[ \gamma \partial _t E^TAP - E^T A\partial _t P \right] + \text{ higher } \text{ time } \text{ derivatives } \text{ of } E^T A P + O(\{E,A,p\}^4) \end{aligned}$$

where $\gamma$ and *T* are constants. We do not include $E^T\partial _t A P$, as it can be absorbed into the two other terms by a partial integration. We define the time scale *T* such that the coefficient of the second term in the integral becomes 1. Using the variational principle, we derive the Euler–Lagrange (EL) equations

11 $$\begin{aligned} \delta _p {\mathcal {L}}: \quad E^T\partial _t A&= -(\gamma +1) \partial _t E^T A,&\delta _E {\mathcal {L}}: \quad A \partial _t P&= -{\gamma \over \gamma +1 } \partial _t A P,&\delta _A {\mathcal {L}}: \quad \gamma \partial _t E_i P_\mu&=E_i \partial _t P_\mu . \end{aligned}$$

where $\delta _X {\mathcal {L}}= {\partial {\mathcal {L}}\over \partial X} - \partial _t {\partial {\mathcal {L}}\over \partial (\partial _t X)} = 0$ for $X\in E,A,P$. A Lagrangian with purely first order time derivatives does not yield any dynamics, as the EL equations will only describe relations among the time derivatives. In reality, response times in the system will result in second order time derivatives, yielding non-trivial dynamics. Motivated by the structure of Eq. (11), we construct a phenomenological model, which attempts to capture the response behavior of investors and markets.

### Stability analysis of the model

Comparing the dynamical Eqs. (6)–(8) with Eq. (11) based on expansion around equilibrium we find approximately $\alpha = -\gamma /(\gamma +1)$ and $\beta = -(\gamma +1)$, hence $\gamma = \alpha \beta$. From Eq. (10) we see that when $\gamma \partial _t E^TAP= E^T A\partial _t P$, the response is no longer linear and we need to consider higher order terms. Taking a mean-field approach, we estimate the critical value of $\gamma$ for a given ratio of the exposure to sovereign debt to equity $\lambda = {\mathbb {E}}[AP/E]$. From Eqs. (8) and (10), we find that the linear response terms vanish when $(\gamma - \lambda ^{-1}) \partial _t P^T A^TAP =0$. This suggests that when $\gamma \lambda = 1$, the system could go through a phase transition. This critical $\gamma$ can also be derived directly from Eqs. (6)–(8) using the mean-field assumption (see Supplementary Section S1). Writing $E\approx AP/\lambda$, we remove the dependence on *E* and *A*, yielding a single equation

12 $$\begin{aligned} \left[ \tau \partial _t^2+\partial _t+\omega ^2 \right] \partial _t P = O\left( (\partial _t P)^2\right) \approx 0, \qquad \text {where }\omega ^2 = {1-\gamma \lambda \over \tau _P+\tau _A}. \end{aligned}$$

This suggests that $\partial _t P$ behaves approximately like a damped oscillator in the mean field model. When $\omega ^2 < 0$, which occurs when $\gamma \lambda >1$, the system becomes unstable. It is worth noting that Eq. (12) is almost identical to the equation proposed in^33^ to describe the behavior of a market near a phase transition, heading from normal behavior toward a crash.

**Figure 2:**
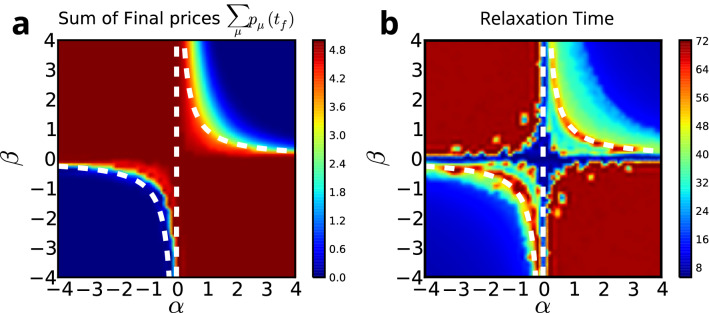
(**a**) The phase diagram of the model using GIIPS sovereign debt data. The colors indicate the sum of the ratios $\sum _\mu P_\mu (t)/P_\mu (0)$ for final asset prices. In one phase (red) the average final price is high, while it drops to zero in the other phase (blue). (**b**) The time the system takes to reach the new equilibrium phase. This relaxation time significantly increases around the transition region akin to a critical slowing down. The white dashed line indicates $\gamma =\alpha \beta =1$, which is close to where the transition occurs.

Figure 2 shows the full phase diagram of Eqs. (6)–(8) and the critical slowing down, simulated on the data set of GIIPS holders in 2011. Our stability analysis of the GIIPS data (Fig. 2a) shows that the transition occurs close to $\gamma = 1$. We also observe that the dynamics slow down dramatically near $\gamma = 1$ (Fig. 2b). This is reminiscent of the critical slowing down in a second order phase transition.

The regimes of stability of the system’s behavior can be summarized as follows:

1. When $\gamma \lambda >1$, shocks cause exponential increases or drops in asset prices and equities. If $\gamma \lambda >1$ persists for an extended period of time, we may observe the formation of a bubble or a crash.
2. When $0<\gamma \lambda < 1$, values of $P_\mu$ and $E_i$ respond to the shock and settle into a new equilibrium state close to the initial state. Bankruptcies are contained and can be explained by bank-specific equity levels $E_i$ and asset holdings $A_{i\mu }$.
3. When $\gamma \lambda <0$, shocks give rise to fluctuations in $P_\mu$ and $E_i$. However, prices and equity levels eventually settle close to their original values.

**Figure 3:**
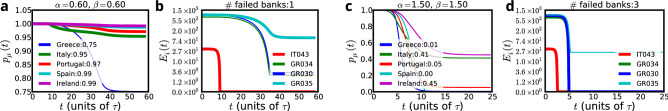
Applying an exogenous shock to the liquid capital of Bank of America. (**a**) Asset prices vs. time for $\alpha =\beta =0.6$. Greek debt incurs the greatest losses, falling to 75% of its original value. The legend shows the final prices. (**b**) Equity of the four most vulnerable banks. Three major Greek banks incur large losses, and one Italian bank is predicted to fail if $\alpha =\beta =0.6$. IT043 is Banco Popolare, which has very small equity but large Italian debt holdings. The other three are Agricultural Bank of Greece, EFG Eurobank Ergasias, and T.T. Hellenic Postbank S.A., all among the top holders of Greek sovereign debt. (**c**) Results for larger values $\alpha =\beta =1.5$. This time also Spanish and Portuguese debt show the next highest level of deterioration. (**d**) The same four banks are the most vulnerable for $\alpha =\beta =1.5$, but this time two more banks fail.

### Stability analysis of the European financial system

In the empirical part of our study, we analyze 119 banks, investment funds, and insurance companies, which represented the largest institutional holders of GIIPS sovereign debt in 2011. (Hereafter, for simplicity, we use the term “banks” to refer to all these financial institutions.) We use the sovereign debt holdings of the banks as their assets $\sum _\mu A_{i\mu }P_\mu$ and use their equity as $E_i$.

As expected, we observe that banks with a very low level of equity fail rapidly in case of a shock; they cease trading and are no longer able to contribute to a decline in asset prices. As a result, they are able to inflict damage on the system only for a brief period of time. We identify four such banks in our data set. However, banks whose equity positions are weak but sufficient to survive a shock cause larger systemic damage. Since they are distressed but able to survive for a significant period of time, they continually exert downward pressure on asset prices. Therefore, banks that are on the cusp of failing but do not fail immediately, have a larger aggregate effect on the financial system than extremely weak banks.

**Figure 4:**
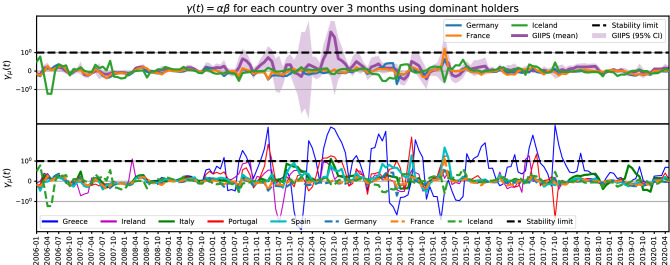
Estimates of $\gamma =\alpha \beta$ over three-month periods. **Top:** the purple curve shows the average of $\gamma _\mu$ over the GIIPS countries and the shaded area indicates 95% confidence interval. **Bottom:** Calculation of $\gamma _\mu$ for individual countries. GIIPS countries are shown as solid curves, and Germany, France and Iceland are shown as dashed line for reference. GIIPS countries show higher values of $\gamma$ which also fluctuated more over time. Since late 2009, $\gamma$ for GIIPS countries has risen close to or above the stability threshold $\gamma =1$, most notably, Greece and Portugal. Before the height of the crisis $0<|\gamma |<1$. It reaches $\gamma > 1$ at the height of the crisis in late 2012. After the crisis, we see the average $\gamma$ decrease again to $\gamma <1$. In contrast, Germany and France stayed in the stable regime for almost the entire period shown.

In our model $\alpha$ and $\beta$ are behavioral parameters and are difficult to measure independently.

The stability of the system in our model depends on the product of the two parameters $\gamma \equiv \alpha \beta$ and $\lambda$. In the data for the dominant holders of GIIPS debt in 2011, the average value for $\lambda$ of the banks was 1.2. We will thus use $\gamma =1$ as an approximate stability bound in our analysis. When $\gamma < 1$, the system is relatively stable with shocks not resulting in catastrophic crashes. Conversely, $\gamma >1$ represents an unstable regime, where positive shocks can create bubbles and negative shocks result in the crash of the entire system^34^. To measure the stability of the eurozone sovereign debt market we simplify Eqs. (6) and (7) to obtain an approximate equation for $\gamma (t)$. Assuming that the time lag $\tau$ is small compared to the time scale over which $\alpha$ and $\beta$ change, we have

13 $$\begin{aligned} \frac{\Delta P_\mu }{P_\mu } \approx \alpha \frac{\Delta A_\mu }{A_\mu }\approx \alpha \beta \sum _{i\in {\mathcal {B}}_{\mathrm {Dom}}} \frac{\Delta E_i}{E_i}\frac{A_{i\mu }}{A_\mu }. \end{aligned}$$

Hence, $\gamma = \alpha \beta$ relates the returns of assets $\Delta P_\mu /P_\mu$ to the percent change on bank equities $\Delta E_i/E_i$ (e.g., common stocks for public banks) weighted by the fraction of total sovereign debt held by the banks, which are the dominant holders $A_{i\mu } /A_\mu$ (see “Methods”).

For $\gamma <1$, when the system is shocked, it reaches a new equilibrium near the initial conditions (Fig. 3a,b). When $\gamma =\alpha \beta \approx 2$, such as at the height of the crisis, even a small shock could have a devastating effect (Fig. 3c,d). Although many banks incur significant losses when $\alpha$ and $\beta$ values are at their highest, the same four banks are severely distressed in both regimes $\alpha =\beta =0.6$ and $\alpha =\beta =1.5$. Figure 3 shows examples of the time evolution of the asset prices and the equity of the banks that incurred the largest losses. Three of the four most vulnerable banks in our simulation are major holders of Greek sovereign debt, which is the asset that has the highest losses, followed by Portuguese debt (Real-world data indicates that the loss on Irish debt was as severe as that on Portugal’s). Our model bases the loss prediction solely on the network of banks which are holding GIIPS sovereign debt.

Assuming herding, we approximate an average $\gamma$ for the GIIPS countries (Fig. 4, top), and also calculate individual $\gamma _\mu$, to examine differences among countries (Fig. 4, bottom). For the eurozone countries from 2006 to 2020 we observe the following:

1. Before the onset of the crisis in 2009, $\gamma$ remained in the stable regime ($\gamma < 1$).
2. Greece entered the unstable regime ($\gamma >1$) numerous times between 2010 and 2020.
3. Portugal and Ireland also reached alarming values of $\gamma$ during the crisis in 2011 and 2012, but were relatively more stable than Greece.
4. Throughout the entire period of 2006-2020, Germany and France mainly remained in the stable regime, with $\gamma$ generally being smaller than one.

These findings are consistent with real-world events, with Greece being the country with the highest volatility in its sovereign debt. Our model’s claim that $\gamma >1$ indicates a potentially unstable regime is further validated by the fact that the GIIPS countries reached highest values of $\gamma$ during the eurozone crisis, while during the same time $\gamma$ for Germany and France remained small. Furthermore, the three countries Greece, Portugal and Ireland with the highest $\gamma$ during the crisis were precisely the three countries that required bail-outs during the crisis, further validating our model’s prediction.

Note that our estimates for 2006-2010 are based on the assumption that the holdings of major sovereign bond holders did not change significantly, as the earliest detailed disclosure of these holdings available to us is from the end of 2011. Additionally, after 2011, we have biannual holdings starting 2014 until 2019, containing only banks supervised by the EBA. Despite these limitations, our results are compatible with the real-world events. Individual plots for $\gamma$ for each country can be found in the supplementary material in Fig. S1. Overall we find that the GIIPS countries had the highest average $\gamma$ among the eurozone countries we examined, though Iceland also reaches values comparable to Italy.

**Figure 5:**
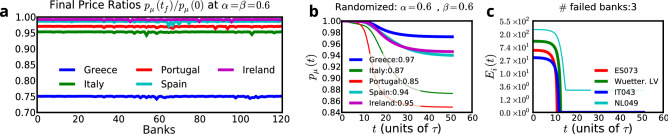
(**a**) Shocking different banks at $\alpha =\beta =0.6$. The final prices of the GIIPS sovereign debts are similar to the original prices. The horizontal x-axis displays bank’s identification numbers from 1 to 119. The vertical y-axis displays asset prices. (**b**) and (**c**) Randomizing which bank lends to which country, while keeping total debt constant for each country. The results differ dramatically from the real-world data used in Fig. 3. In this example Portuguese and Italian sovereign debts experience largest losses, while Greek debt is the least vulnerable. Other random realizations yield different results.

### Impact of the network structure

To demonstrate the importance of the underlying network structure, we examine the effect of random rewiring between banks and sovereign debt. Our goal is to determine to what extent the dynamics of the system are caused by the network structure. To this end, we perform five different randomizations of the network $A_{i\mu }$:

1. Weight shuffled: we only shuffle the weights while keeping the structure of the links.
2. Unweighted: we keep all nonzero links and assign them a weight equal to the average of *A*.
3. Randomizing banks: we keep the total outstanding debt for each country the same and randomize the holders.
4. Randomizing sovereign debt: we keep the total bank exposure unchanged and modify the origin of sovereign debt.
5. Complete randomization: we shuffle all links.

For the sovereign debts, we observe that any randomization changes the vulnerability of each country, emphasizing the importance of the network structure and the weights. Greek debt, for example, is no longer the most vulnerable when the network is randomized. For the banks, however, we observe that some banks, including the National Bank of Greece and Piraeus Bank, consistently rank among the top five most vulnerable banks for almost all randomizations. This suggests that the level of equity for these banks is too low for them to survive a shock.

Figure 5 shows an example of network randomization and how dramatically the final results differ. The randomized network demonstrates two important features of the model: (i) system dynamics are strongly affected by the network structure, i.e., knowing only variables such as the equity and exposure of individual banks is not sufficient, and (ii) real-world data seems to indicate that the composition of the holders of Greek sovereign debt affects the value of the bonds.

The vulnerability of sovereign debt is affected by the stability of a country’s government, and the greater the stability of a government, the lower the risk of the sovereign debt. Predominantly, banks hold domestic sovereign debt, leading to a lack of diversification in their portfolios. Since we are building the network based on these holdings, the topological features capture this home bias. We observe in our simulations that this greatly impacts the outcomes after a shock. If these holdings are distributed differently to eliminate the inherent home bias, the vulnerability of the financial system is reduced as both the sovereign debt and the banks are less affected by a downturn in their domestic economy. Similarly, if the amounts of sovereign debt holdings are distributed differently without changing the ownership structure, this also improves the stability. The detailed results for all the randomizations are shown in Fig. S2.

### BankRank as a measure of systemic impact

Based on our observations above, firms at the threshold of failure exhibit the largest threat to the system. Therefore, we define a “survival equity ratio” which is the minimum fraction of the equity a bank needs to survive a shock. At the same time, this ratio is also the one at which a bank can induce the most damage to other banks. Consider, for example, a well-capitalized bank with $1bn in equity when a shock enters the system through other banks. Based on its holdings and portfolio overlap with other financial institutions, the bank is affected by the shock, but it survives. By how much could we reduce the bank’s equity and still avoid its failure? If the bank were to fail as soon as the equity dropped below $0.8bn after a shock hits the system then the “survival equity ratio” would be 0.8 or 80 percent. A bank at the cusp of failure can cause varying degrees of damage, which differs significantly from bank to bank. The reason for this lies in the structure of the financial network arising from the banks’ portfolios. Understanding the effect of individual financial institutions on the vulnerability of the entire network is an important input into creating effective strategies for mitigating the systemic risk^35^. In order to assess the potential impact of each bank, we create a scenario in which the banks’ equity levels are at their survival equity ratios when we stress the financial network.

We analyze two scenarios. In both cases, we start with the empirical network of European banks and assets. In the first scenario, we increase the equity of the four banks that we have identified as failing to their survival equity ratio: ${\tilde{E}}_{i}(0)=\sum _{\mu } A_{i\mu }(0)P_{\mu }(0)$. We leave the equity of the other banks unchanged. This procedure makes the system resilient to shocks when $\gamma =\alpha \beta <1$, that is, when the combination of panic factor and asset illiquidity does not exacerbate a shock. In this regime, the decrease in sovereign debt prices stays below 1% by the time the system reaches its equilibrium. In the unstable regime, however, where $\gamma >1$ the system incurs significant losses from the negative effect of the banks that are on the cusp of failure and could propagate value loss throughout the financial network.

In the second scenario, we assess the systemic importance of each bank one by one. We numerically determine the survival equity $E^*_i$ for which the bank does not fail such that for $E_{i}(0)<E^*_i$ the bank fails. We then set the initial condition for bank *i* to this critical value of equity, ${\tilde{E}}_{i}(0)=E^*_i$. To induce stress to the system, we shock one bank $j\ne i$, selected from the banks in the system. Our results are independent of the source of the shock, as Fig. 5 illustrates. However, regardless of the origin of the shock *j*, the contagion process plays out differently for each bank *i* because it has uniquely different sovereign debt holdings, $\sum _\mu A_{i\mu }P_\mu$. We introduce a network measure to use the variations in the $A_{i\mu }$ to construct BankRank, which describes the different level of systemic importance of each bank.

We define *BankRank* of bank *i* as the ratio of remaining value of assets in the system at the final time step $t=t_f$ over the initial holdings at time $t=0$ when ${\tilde{E}}_i=E^*_i$. Therefore, a smaller value of $R_i$ indicates a greater the systemic importance of bank *i*:

14 $$\begin{aligned} {\mathbf{BankRank}} \, of \, Bank \, i:R_i= \frac{\sum _\mu \sum _j A_{j\mu }(t_f)P_{\mu }(t_f)}{\sum _\mu \sum _j A_{j\mu }(0)P_{\mu }(0)}\biggr |_{{\tilde{E}}_i=E^*_i}. \end{aligned}$$

**Figure 6:**
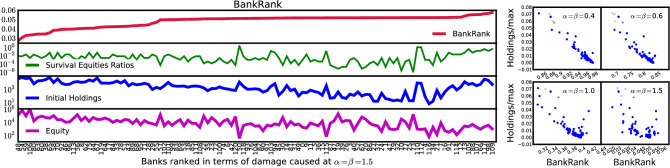
**Left:** We show the systemic importance of banks according to their BankRank, indicating the damage their failure inflicts on the entire system. Each bank is denoted by a number; refer to Table S3. for their respective names. The second plot shows the survival equity ratio $E^*/{\tilde{E}}$, the third is the initial holdings, and the fourth shows the initial equity, all sorted in terms of BankRank at $\alpha =\beta =1.5$. As we see, none of these three variables correlates highly with BankRank. The ranking changes for different values of $\alpha$ and $\beta$. **Right:** Scatter plot of the holdings divided by maximum holdings (Holdings/max) on y-axis vs. BankRank on x-axis for four different values of $\alpha =\beta =[0.4,0.6,1,1.5]$. As we see, increasing $\alpha \beta$ decreases the correlation between BankRank and initial holdings. BankRank at $\gamma =\alpha \beta <1$ is strongly negatively correlated with the holdings. But at $\gamma =\alpha \beta >1$, BankRank deviates significantly from the holdings. In the unstable regime $\gamma >1$ it is no longer true that only the largest holders have the highest systemic importance.

### Comparison of BankRank to portfolios

In Fig. 6 we show BankRank in the unstable regime at $\alpha =\beta =1.5$ and how it compares to (1) survival equity ratio $E^*_i/E_i$, the ratio of minimum equity required for survival and initial equity, (2) initial holdings, and (3) bank’s equity. We observe some correlation between BankRank and each of these variables. In Fig. 6, we show a comparison of BankRank and the bank’s holdings, which represents the banks’ degree centrality in the network. In the stable regime where $\alpha \beta <1$ the correlation is high; in calm and liquid financial markets, the size of the portfolio seems to be a good proxy for the systemic importance. However, in the unstable regime where $\alpha \beta >1$ the correlation between the BankRank and bank holdings is much weaker and actually insignificant ($p=0.52$). Therefore, while in the stable regime sovereign debt holdings almost completely determine the systemic importance of a bank, in the unstable regime smaller holders might have high systemic importance. We further compare BankRank in the unstable regime with various centrality measures^16^ (Fig. S3) and find no significant correlations with eigenvector centrality ($p=0.52$), closeness centrality ($p=0.60$) and flow betweenness centrality ($p=0.40$). To calculate these centrality measures, we construct a bank-bank exposure network $B= AA^T$ from the holdings matrix *A*. Note that none of the above familiar network centrality measures take the equity of banks into account, whereas BankRank is sensitive to the equity of banks. Given the insignificant correlation of BankRank with the familiar centrality measures, we believe that BankRank in the unstable regime captures an important dynamical property of this system. Since banks with a very small “survival equity ratio” are able to absorb a large initial shock, the combination of BankRank and this ratio can inform stress tests for individual banks.

## Discussion

We introduce a model for studying the systemic importance of investors in a financial market as well as assessing the stability of the market. We apply our model to the large institutional holders of GIIPS sovereign debt and investigate their impact on systemic risk at the height of the European sovereign debt crisis of 2009-2012. We analyze the period surrounding the crisis and contrast the stability during the crisis versus the non-crisis period. Our methodology can be used to model systemic risk propagation through a bipartite network of banks and assets, i.e., the model can serve as a “systemic stress testing” tool for complex financial systems, and it can be used to identify the stability of a financial network.

Specifically, our model predicts that a parameter $\gamma$ relating the returns on assets to the percent change in the equity of the investors is a good indicator of the stability of the market. We predict that the market is unstable when $\gamma >1$, while when $\gamma <1$ the system is stable. The predictions of our model regarding the stability of the GIIPS holders network match well the time-line of the European sovereign debt crisis.

We also propose a simple, dynamic systemic risk indicator, BankRank, which measures the amount of damage that the bank network suffers from a failure of a particular bank. While the system is in a stable state, BankRank has significant correlation with GIIPS sovereign debt holdings, while in the unstable state BankRank doesn’t correlate well with initial sovereign debt holdings or equity of banks. This shows that simple measures such as initial size of the bank or distribution of bank assets cannot determine the systemic importance of banks. Our method improves the measurement of systemic risk in bank networks by investigating the significance of the network structure and proposing that the relations among banks through shared portfolios are central for assessing the risk of the banking system. Our model may serve as a monitoring and simulation tool for policymakers to identify systemically important financial institutions and to assess systemic risk build-up in financial networks.

Real-time monitoring of the accumulation of risk in the financial system could improve risk management by central bankers, as well as by financial institutions themselves. Policymakers could be better equipped to identify the sources of risk and propose policies that could increase the stability of the entire system. Understanding the complexities and the interconnectivity of the financial system is a first step to taking preventative measures and protecting the financial network from catastrophic failures with lasting consequences. Lastly, our view is that the model we propose in this paper, while tested on the European sovereign debt crisis, is suitable for other countries and regions of the world, as well as other sources of risk.

## Methods

### Data set

We start with a data set covering 136 banks, investment funds, and insurance companies, which represent the largest institutional holders of GIIPS sovereign debt in 2011. (Hereafter, for simplicity, we use the term “banks” to refer to all these financial institutions.) Table 2 shows the percentages of the sovereign bonds issued by each GIIPS country owned by these banks. Since our model requires knowledge of the equity of each bank, we reduce our data set to those 121 banks for which we could obtain this information. By the end of 2011, two important Greek banks – the National Bank of Greece and Piraeus Bank – had negative equity. Because our model only considers banks that can execute trades based on positive equity, we also eliminate these two banks from our analysis.

**Table 2:** Total amount of exposure of the banks in our data set to the sovereign debt of the GIIPS countries in 2011.

	Greece	Italy	Portugal	Spain	Ireland
Total (bnEu)	273.96	1641.49	128.63	692.98	89.58
Data set 2011 (bnEu)	96.90	420.55	48.93	333.46	32.60
% in banks	35.37	25.62	38.04	48.12	36.39

Due to changes in regulation and policy, the EBA modified its framework for bank stress testing after 2011 and introduced a “Transparency Exercise” in which banks report their holdings semiannually. To account for name changes and restructurings in the period from 2014 to 2019, we have consolidated the data accordingly. Consolidated entries are marked as such in the supplementary material. The number of banks in the data set fluctuates year over year, and it is at its lowest at 87 in 2015/16 and at its highest at 126 in 2019.

From our data set, we extracted the individual banks’ gross exposures to GIIPS sovereign debt. We aggregated the debt on the banks’ balance sheets across all maturities. For each semiannual report in the Transparency Exercise, we determined the dominant holders according to the following rule: We sorted the banks in order of their percentage holdings of the debt of each of the GIIPS countries from high to low. We then selected the largest holders until the banks in our selection make up at least 2/3 of the total holdings for the respective country. If our selection consisted of less than four banks or we did not have stock price information for at least four banks, we supplemented the selection by the next largest bank(s) until we had at least four banks for which we could compute equity returns. Please refer to the supplementary Table S1 for the list of dominant GIIPS holder at the end of 2011 and 2019 and Table S2 for the names of the banks corresponding to the above ticker symbols. Bank equity is mostly comprised of the shareholders’ equity, or common stock, which we approximated by the market value of equity. We estimated $\Delta E_i/ E_i$ as the ordinary return of the stock of these dominant sovereign debt holders. We retrieved their stock prices from Yahoo Finance, using the monthly adjusted close prices in euros. To compute the change in bond prices, we obtained the monthly yield of 10-year bonds for the GIIPS countries from the Federal Reserve Economic Database (FRED).

### Parameter estimation

We estimate the values of our parameters for the GIIPS sovereign debt crisis. We use approximate versions of the differential Eqs. (6) to (8) in order to estimate $\gamma =\alpha \beta$. The distribution of asset holdings is roughly log-normal; as a result, a large portion of each GIIPS country’s debt is held by only a few banks. Therefore, using the equity of the dominant holders of the debt $\mu$ accomplishes a good estimate of $\gamma$. We denote this set of dominant holders ${\mathcal {B}}_{\mathrm {Dom}}$.

We further estimate that the response time $\tau$ is at most on the order of several days. Therefore, we calculate $\gamma =\alpha \beta$ over a period of four months to allow the system to reach its new final state. From Eq. (4) we have

15 $$\begin{aligned} \frac{\Delta A_{\mu }}{A_\mu } \approx \beta \sum _{i\in {\mathcal {B}}_{\mathrm {Dom}}} \frac{\Delta E_i}{E_i}\frac{A_{i\mu }}{A_\mu } \end{aligned}$$

where the ${A_{i\mu }/ A_\mu }$ factor ensures that we have a weighted average of returns $\Delta E_i/E_i$ based on how large banks’ holdings are. Since we are performing our estimation for the dominant holders, for consistency we compute $A_\mu =\sum _{i\in \mathrm {Dom}} A_{i\mu }$, weighting only using dominant holders. Using this approximation, we can relate the first two equations,

16 $$\begin{aligned} \frac{\Delta P_\mu }{P_\mu } \approx \alpha \frac{\Delta A_\mu }{ A_\mu }\approx \alpha \beta \sum _{i\in {\mathcal {B}}_{\mathrm {Dom}}} \frac{\Delta E_i}{E_i}\frac{A_{i\mu }}{A_\mu }. \end{aligned}$$

We approximate the product $\gamma \equiv \alpha \beta$ using linear regression. To examine the validity of our assumption about herding, we calculate separate $\gamma _\mu (t)$ for each country $\mu$. We estimate the returns on sovereign bonds $\Delta P_\mu /P_\mu$ from the yield time series using the duration-with-convexity rule. For simplicity, we assume that aggregate duration and convexity of the sovereign bonds are 10 and 100, respectively. We further compute returns of common stocks of major holders as the percentage change in the stock price, and weight it by the fraction of their holdings $A_{i\mu }/A_\mu$. Since sovereign debt holdings are long-term investments and reported once or twice a year, we use stock and bond return data over three months to calculate $\gamma _\mu (t)$ (returns spanning $[t-3,t]$ months) as follows:

17 $$\begin{aligned} \delta P_\mu (t)&\equiv {\Delta P_\mu (t)\over P_\mu (t) },\qquad \delta E_\mu (t) \equiv \sum _{i\in {\mathcal {B}}_{\mathrm {Dom}}} \frac{\Delta E_i(t)}{E_i(t)}\frac{A_{i\mu }(t)}{A_\mu (t)} ,\qquad&\delta P_\mu (t')&= \gamma _\mu (t) \delta E_\mu (t'), \quad t' \in [t-3,t] \end{aligned}$$

18 $$\begin{aligned} \gamma _\mu (t)&= \frac{\sum _{t'=t-3}^t\delta P_\mu (t') \delta E_\mu (t')}{\sum _{ t'=t-3}^t\delta E_\mu ^2(t')},&\end{aligned}$$

19 $$\begin{aligned} \gamma (t)&= \frac{\sum _\mu \sum _{t'=t-3}^t\delta P_\mu (t') \delta E_\mu (t')}{\sum _\mu \sum _{ t'=t-3}^t\delta E_\mu ^2(t')},&\end{aligned}$$

We evaluate $\gamma$ for the time period between 2006 to 2019 and observe stable and unstable regimes related to the eurozone crisis. Fig. 4 shows the parameter estimates for $\gamma$. For a more detailed overview of the parameters see Fig. S1.

## Supplementary Information

Supplementary Information 1.

Supplementary Information 2.
