# Supplementary material for: Systemic stress test model for shared portfolio networks
Source: Sci Rep. 2021 Feb 8;11:3358. doi: 10.1038/s41598-021-82904-y (PMC7870944; doi:10.1038/s41598-021-82904-y)
Supplement: Supplementary file 2 — Supplementary Information 2. [file 41598_2021_82904_MOESM2_ESM.pdf]

Supplementary Material

Systemic stress test model for shared portfolio networks

[1,2,*]**Irena Vodenska** [2]**Nima Dehmamy** [1,2]**Alexander P. Becker** [3]**Sergey V. Buldyrev** [4]**Shlomo Havlin**

[1]Department of Administrative Sciences, Metropolitan College, Boston University, 1010 Commonwealth Avenue, Boston, MA 02215, USA [2]Center for Polymer Studies and Department of Physics, Boston University, 590 Commonwealth Avenue, Boston, MA 02215, USA [3]Department of Physics, Yeshiva University, 500 West 185th Street, New York, NY 10033, USA [4]Bar-Ilan University, 52900 Ramat-Gan, Israel [*]vodenska@bu.edu

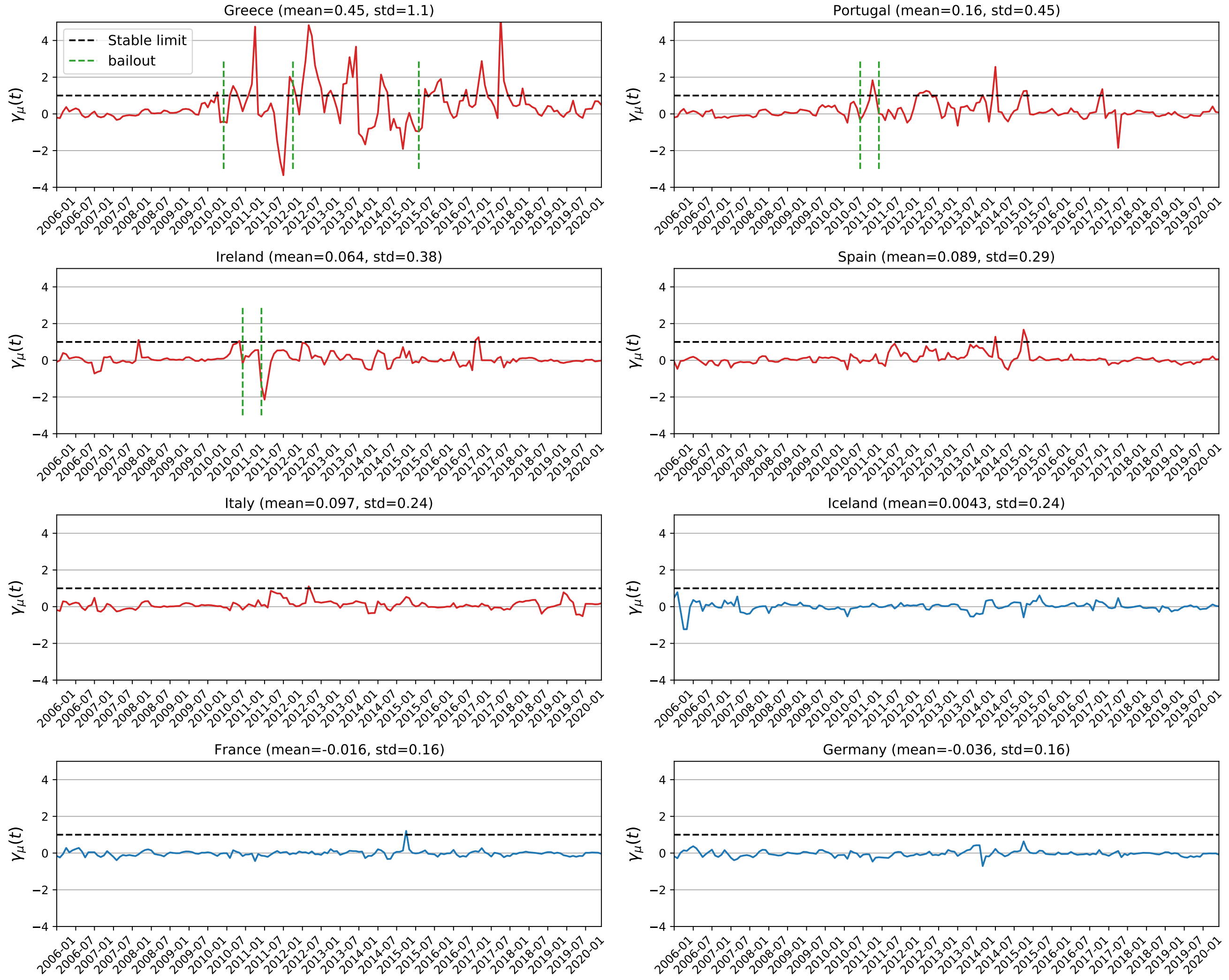

Figure S1. $\gamma_\mu(t)$ calculated for each country based on their dominant holders, over three-month sliding windows. The countries are sorted from highest to lowest mean plus one standard deviation of gamma. All GIIPS countries (red curves) have the largest γ, though the values for Italy are close to Iceland. France and Germany exhibit significantly smaller mean and standard deviation in γ, which we interpret as indicating a more stable economy. The bailout dates for Greece, Ireland and Portugal are shown as vertical green dashed lines.

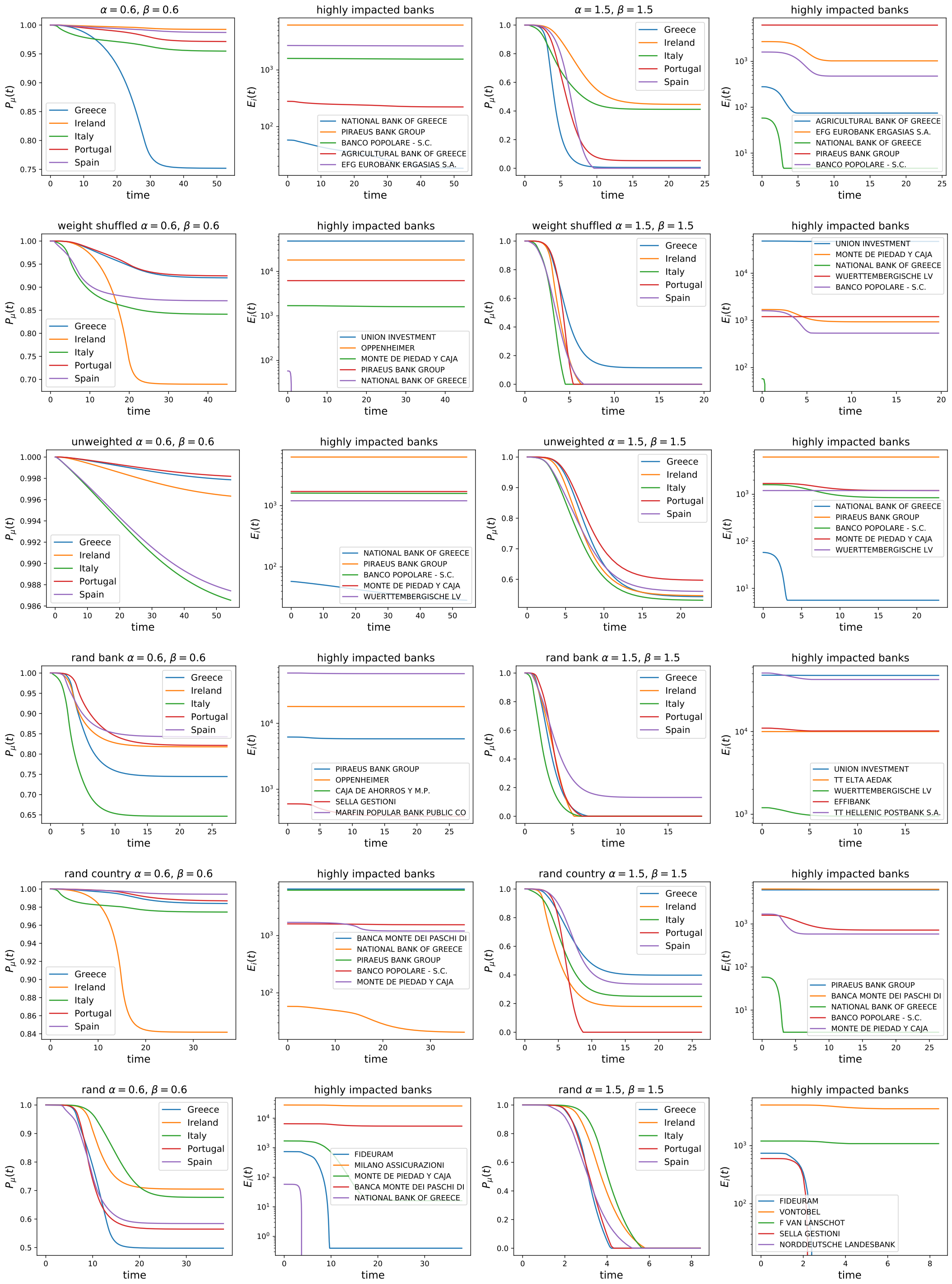

Figure S2. Effect of various randomizations of the network. From top to bottom, we show sample results for the following: no randomization for reference; weight shuffled randomization in which we only shuffle the weights while keeping the structure of the links; unweighted randomization where we keep all nonzero links and assign them a weight equal to the average of A; randomizing banks where we keep the total outstanding debt for each country the same and randomize the holders; randomizing sovereign debt in which we keep the total bank exposure unchanged and modify the origin of sovereign debt; and complete randomization where we shuffle all links. Most randomizations we tried resulted in a change of which countries and banks are vulnerable. This suggests that both the topology, as well as the weights of the network are crucial for predicting vulnerabilities.

Table S1. List of tickers for the dominant holders of GIIPS debt in the end of 2011 and 2019.

Country	Year	Tickers
Greece	2011	BNP.PA, ALPHA.AT, EUROB.AT, TPEIR.AT, ETE.AT
	2019	TPEIR.AT, EUROB.AT, ETE.AT, ALPHA.AT
Ireland	2011	G, MUV2.MI, BEN, AIBG.L, BIRG.IR, IL0A.IR, GLE.PA
	2019	BIRG.IR, AIBG.L, BNP.PA, DANSKE.CO
Italy	2011	UCG.MI, BNP.PA, ISP.MI, BAMI.MI, BMPS.MI, ALV.DE, UBI.MI, UNI.MI, CBK.DE, ACA.PA, CS.PA
	2019	UCG.MI, ISP.MI, BNP.PA, BMPS.MI, BAMI.MI, CBK.DE, ACA.PA
Portugal	2011	G, SAN, BNP.PA, BBPI.LS, BCS, BCP.LS, CS.PA
	2019	SAN, SAB.MC, BNP.PA, BCP.LS
Spain	2011	G, SAN, SAB.MC, ALV.DE, BCS, BBVA.MC, BKIA.MC, CS.PA
	2019	BBVA.MC, SAN, SAB.MC, UCG.MI, ISP.MI, BNP.PA

S1 Comparison of BankRank in the unstable regime with other centrality measures

We found that BankRank in the unstable regime (e.g. $\alpha = \beta = 1.5$) did not correlate well with holdings, unlike the in the stable regime. We further compare BankRank in the unstable regime with other centrality measures to see if BankRank is truly capturing a different property of the system. Most centrality measures such as eigenvector, closeness and betweenness centrality are defined for monopartite networks. In order to compare BankRank of the banks with these cetrality measures, we first construct a monopartite Bank-Bank network with adjacency $B = AA^T$ where $A_{i\mu}$ are the elements of the matrix of bank holdings. We then compute the degree, eigenvector, closeness and betweenness centrality on B and calculate the Spearman rank correlation of these centrality measures with the BankRank calculated at $\alpha = \beta = 1.5$. The results, shown in Fig. S3 show that

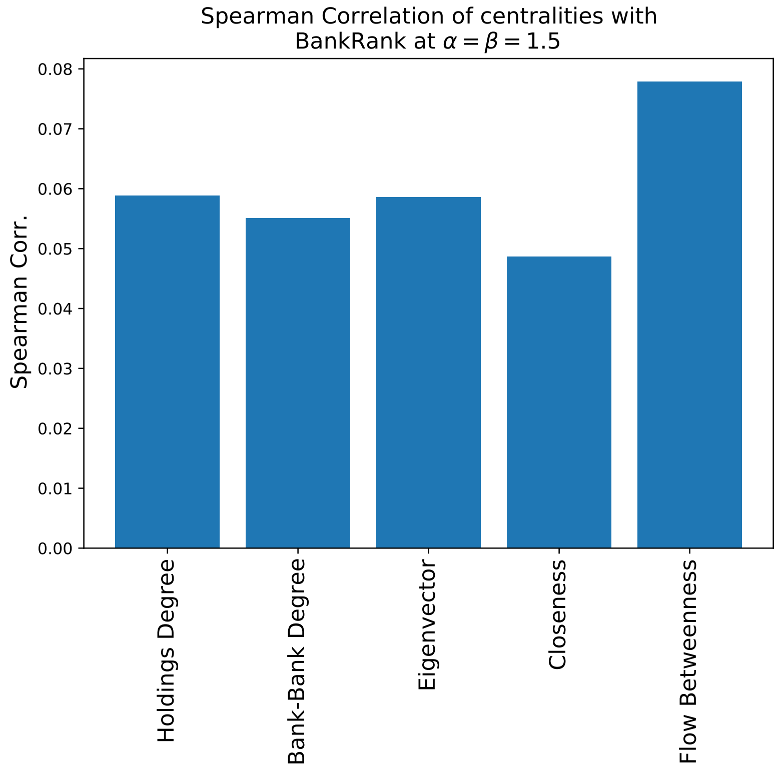

Figure S3. Spearman Rank Correlation of BankRank in the unstable regime ($\alpha = \beta = 1.5$) with various centrality measures. None of the measures show a significant correlation with BankRank.

none of these centrality measures has considerable correlation with BankRank in the unstable regime. This lack of correlation is expected as BankRank in the unstable regime is sensitive to both the holdings as well as the equities of the banks, whereas all of these centrality measures only use the holdings and do not incorporate the bank equities.

Table S2. List of the dominant holders of GIIPS sovereign debt in the 2011 and 2014-2019 data sets and their ticker symbols.

Bank Name 2014-2019	Bank Name 2011	Ticker	Currency
ALLIED IRISH BANKS (C)	ALLIED IRISH BANKS PLC	AIBG.L	GBP
ALPHA BANK, S.A.	ALPHA BANK	ALPHA.AT	EUR
BANCA MONTE DEI PASCHI DI SIENA S.P.A.	BANCA MONTE DEI PASCHI DI	BMPS.MI	EUR
BANCA POPOLARE DI SONDRIO, SOCIETÀ COOPERATIVA...	-	BPSO.MI	EUR
BANCO BILBAO VIZCAYA ARGENTARIA, S.A.	BBVA	BBVA.MC	EUR
BANCO BPI SA	BANCO BPI SA	BBPI.LS	EUR
BANCO BPM (C)	BANCO POPOLARE - S.C.	BAMI.MI	EUR
BANCO COMERCIAL PORTUGUÊS, SA	BANCO COMERCIAL PORTUGUES	BCP.LS	EUR
BANCO DE SABADELL, S.A.	BANCO DE SABADELL S.A.	SAB.MC	EUR
BANCO SANTANDER, S.A.	BANCO SANTANDER S.A.	SAN	USD
BANK OF IRELAND (C)	BANK OF IRELAND	BIRG.IR	EUR
BARCLAYS PLC	BARCLAYS PLC	BCS	USD
BFA-BANKIA	BFA-BANKIA	BKIA.MC	EUR
BNP PARIBAS	BNP PARIBAS	BNP.PA	EUR
BPER BANCA S.P.A.	-	BPE.MI	EUR
CAIXABANK	-	CABK.MC	EUR
COMMERZBANK AKTIENGESELLSCHAFT	COMMERZBANK AG	CBK.DE	EUR
CRÉDIT AGRICOLE GROUP (C)	CREDIT AGRICOLE	ACA.PA	EUR
DANSKE BANK A/S	DANSKE BANK	DANSKE.CO	DKK
DEUTSCHE BANK AG	DEUTSCHE BANK AG	DB	USD
EUROBANK ERGASIAS, S.A.	EFG EUROBANK ERGASIAS S.A.	EUROB.AT	EUR
HSBC HOLDINGS PLC	HSBC HOLDINGS PLC	HSBC	USD
INTESA SANPAOLO S.P.A.	INTESA SANPAOLO S.P.A	ISP.MI	EUR
LIBERBANK, S.A.	-	LBK.MC	EUR
MEDIOBANCA – BANCA DI CREDITO FINANZIARIO S.P.A.	-	MB.MI	EUR
NATIONAL BANK OF GREECE, S.A.	NATIONAL BANK OF GREECE	ETE.AT	EUR
PERMANENT TSB GROUP HOLDINGS PLC	IRISH LIFE AND PERMANENT	IL0A.IR	EUR
PIRAEUS BANK, S.A.	PIRAEUS BANK GROUP	TPEIR.AT	EUR
STATE STREET EUROPE HOLDINGS GERMANY S.A.R.L. ...	STATE STREET	STT	USD
UNICREDIT S.P.A.	UNICREDIT S.P.A	UCG.MI	EUR
UNIONE DI BANCHE ITALIANE S.P.A.	UNIONE DI BANCHE ITALIANE SCPA	UBI.MI	EUR

S2 Analytical derivation of the mean-field phase space

Figure 3 shows an example of the average final prices and the time the system needs to reach its final state for various values of α and β. The system exhibits two prominent phases: one in which a new equilibrium is reached without a significant loss in asset value (upper left and lower right quadrants), and another in which the assets lose significant value (above dashed line in the upper right quadrant and lower left quadrant). At the transition in both the first and the third quadrants, the system requires a long time to reach its new equilibrium. Such a behavior in the relaxation time may signal the existence of a second order phase transition, described by $\gamma = \alpha\beta = 1$.

To obtain exact solutions for this phenomenological model, we simplify the equations (6)-(8). We assume that there is one major holder of each asset μ and reduce the system to the interactions of one bank with equity E and holdings A and one asset with price p. With this mean field assumption, we break the network apart and analytically derive the phase transition for this simplified model. As we show below, the 1-by-1 system exhibits the same phases, even if it does not have the richness and complexity of the entire system.

Analytical results from the system with one bank and one asset. In the following, we present the analytical solution of the 1-by-1 model and derive the curve along which the phase transition is happening in Figure 3. The equations for a 1-by-1 system simplify to

$$\frac{(\partial_t + \tau_A \partial_t^2)A}{A} = \beta \frac{\partial_t E}{E} = \beta \frac{A\partial_t P}{E}, \tag{20}$$

$$\frac{(\partial_t + \tau_P \partial_t^2)P}{p} = \alpha \frac{\partial_t A}{A}. \tag{21}$$

We eliminate A and E, and to this end, we find an expression for $\partial_t^2 A/A$. Taking another derivative in equation (21) yields

$$\frac{(\partial_t^2 + \tau_P \partial_t^3)P}{p} - \frac{(\partial_t + \tau_P \partial_t^2)P\partial_t P}{P^2} = \alpha \frac{\partial_t^2 A}{A} - \alpha \left(\frac{\partial_t A}{A}\right)^2. \tag{22}$$

Combining this result with the equation (20) results in:

$$\begin{aligned} \frac{(\partial_t + \tau_P \partial_t^2)P}{P} + \tau_A \frac{(\partial_t^2 + \tau_P \partial_t^3)P}{P} &= \gamma \frac{A\partial_t P}{E} + O\left((\partial_t P)^2\right) \\ \left[\tau_P \tau_A \partial_t^2 + (\tau_P + \tau_A)\partial_t + \left(1 - \gamma \frac{AP}{E}\right)\right] \partial_t P &= PO\left((\partial_t P)^2\right), \end{aligned} \tag{23}$$

where the nonlinear term is again quadratic in P and thus a generalized form of the Fisher equation. More specifically,

$$O\left((\partial_t P)^2\right) = \tau_A \frac{(1 + \tau_P \partial_t)\partial_t P \partial_t P}{p} - \alpha \tau_A \frac{((1 + \tau_P \partial_t)\partial_t P)^2}{p} \tag{24}$$

Next, we show that in the stable regime the nonlinearity in the frequency, that is, the $\gamma AP/E = \gamma\lambda$ term, is of the order $O(\partial_t A \partial_t P)$ and thus remains small if we show that at small times the behavior of $\partial_t P$ in the stable regime is oscillating around zero.

In this regime, the dynamics are richer, and we have a damped oscillator with a driving force coupled to P and nonlinearities of type $(\partial_t P)^2$. Taking the price change $u \equiv \partial_t P$ as the fundamental variable, the nonlinearities are roughly of type $u^2 + a\partial_t u^2$. In short, the equations are

$$\begin{aligned} \left[\tau \partial_t^2 + \partial_t + \omega^2\right] u &= O\left(u^2, \partial_t u^2\right) \\ \frac{1}{\tau} = \frac{1}{\tau_A} + \frac{1}{\tau_P}, \quad \omega^2 &= \frac{1 - \gamma\lambda}{\tau_A + \tau_P}. \end{aligned} \tag{25}$$

Although ω^2 depends on A, P and E, we can use an approximate time-dependent exponential ansatz $u \sim u_0 \exp[\lambda t]$. The solutions to η are:

$$\eta_{\pm} = \frac{-1 \pm \sqrt{1 - 4\tau\omega^2}}{2\tau}.$$

When $\omega^2 > 0$ and $1 - 4\tau\omega^2 < 0$, there will be oscillatory solutions. One such example arises when $\gamma\lambda < -1$, which only happens for negative γ. This is consistent with the simulations which showed that the oscillatory behavior was in the $\alpha\beta < 0$ quadrants. For the stability, however we care about the real solutions.

When $\omega^2 < 0$, which happens when $\gamma\lambda > 1$, we have two real solutions with opposite signs. The presence of the positive root signals an instability because the solution diverges. For a delta function shock of magnitude f at $t = 0$ we find that:

$$E_0 \to E_0(1+f).$$

Having initially scaled to $E_0 = A_0 = P_0 = 1$, the condition for existence of the positive root becomes:

$$t = 0 : \quad \gamma > \lambda^{-1} = (1+f).$$

Now the question is, to which solution does the system trend when it is shocked. The price change $\partial_t P$ is

$$\partial_t P(t) = u(t) = u_+ e^{\eta_+ t} + u_- e^{\eta_- t}$$

Since at $t = 0$ the initial conditions dictated $\partial_t P(0) = 0$, we have

$$u_+ = -u_-.$$

Therefore, both solutions are equally likely. It follows that whenever one of the solutions (u_- in our case) is positive, the solution diverges. When $f > 0$ a bubble forms and grows exponentially and when $f < 0$, because our variables are non-negative, the price crashes to zero. This proves that the sufficient condition for stability is $\gamma < 1$. Further note that the all nonlinear terms are proportional to $\partial_t P$, and therefore, at $t = 0$,

$$O\left(u^2(0), \partial_t u^2(0)\right) = 0.$$

Thus, the solution is exact at $t = 0$ and we get three regimes:

1. When $\omega^2 > \frac{1}{4\tau}$, there will be oscillatory solutions. This happens when $\gamma < \frac{-(\tau_P - \tau_A)^2}{\tau_P \tau_A}$. For $\tau_P = \tau_A$ this is just the $\gamma < 0$ condition we observed for oscillations in our simulations.
2. When $\frac{1}{4\tau} > \omega^2 > 0$, i.e., $\frac{-(\tau_P - \tau_A)^2}{\tau_P \tau_A} < \gamma < 1$, we have decaying solutions but both $\eta_\pm < 0$. Therefore the changes won't be large and eventually the sytems settles in new equilibrium.
3. When $\omega^2 < 0$, i.e., $\gamma > 1$ we will have two real solutions for with opposite signs. The presence of the positive root $\eta_+ > 0$ signals an instability because this solution diverges.

Thus we have proven the existence of the three phases we had observed earlier and derived the transition conditions analytically.

Validity of perturbation theory near the phase transition. For the above solution to be valid we must confirm that the corrections are small. We must find a small parameter that exists in the neglected terms which allows perturbative solutions to be viable. We have two sets of nonlinearities: (1) $O\left((\partial_t P)^2\right)$; (2) $\gamma AP/E$.

- The nonlinearity $O\left((\partial_t P)^2\right)$: Note that the instability occurs when the larger root η_- becomes positive. Therefore, near the transition we have

$$\begin{aligned} &4\tau\omega^2 \ll 1 \\ &\eta_+ \approx -\frac{1}{\tau} + \omega^2 \\ &\eta_- \approx -\omega^2 \end{aligned} \tag{26}$$

  Thus, being close to the phase transition means $\eta_- \ll 1/\tau$. As a consequence, for $O\left((\partial_t P)^2\right)$ and using the $u_+ = -u_-$ found above, we get:

$$\begin{aligned} (\tau_P \partial_t) u =& \tau_P u_+ \left(\eta_+ e^{\eta_+ t} - \eta_- e^{\eta_- t}\right) \\ \approx& \tau_P u_+ \left(\eta_+ e^{\eta_+ t} - \eta_- e^{\eta_- t}\right) \\ O\left(u^2\right) =& \tau_A \frac{u(1+\tau_P \partial_t)u}{p} - \alpha\tau_A \frac{((1+\tau_P \partial_t)u)^2}{p} \\ \approx& \tau_A \frac{u(1+\tau_P u_+(\eta_+ - \eta_-))u}{p} - \alpha\tau_A \frac{((1+\tau_P \partial_t)u)^2}{p} \end{aligned} \tag{27}$$

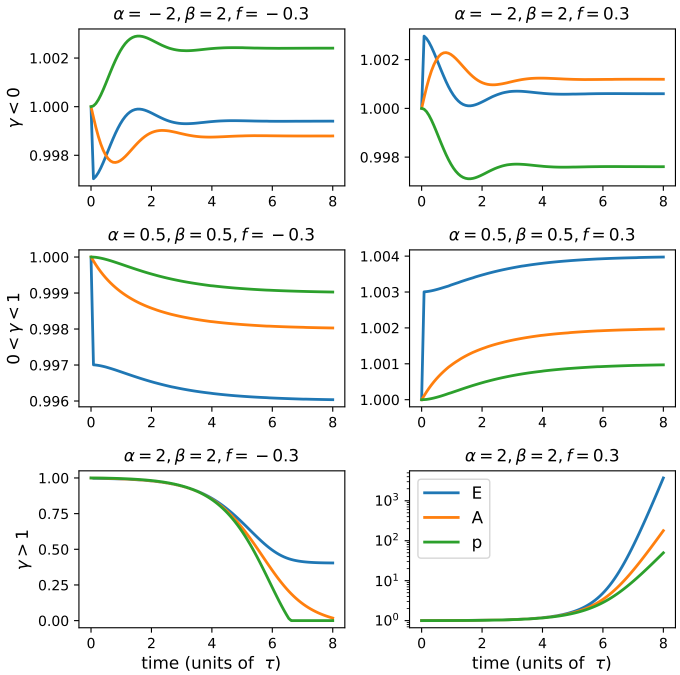

Figure S4. Dynamics of the model for a system with one bank and one asset with $E(0) = A(0) = P(0) = 1$. Here f indicates the magnitude of the shock, which can be positive or negative. In the top row, $\gamma < 0$. Following a the shock, the variables of the system fluctuate for some time and eventually return to their original place or somewhere close. In the middle row, $0 < \gamma < 1$. A shock may result in a non-negligible change to the variables of the system, and this change increases as $\gamma \to 1$. In the bottom row, $\gamma > 1$. A negative shock results in a collapse, and positive shocks results in the formation of a bubble. Either way, the system is unstable.

- The nonlinearity $\gamma AP/E$: We next have to examine if the assumption that $\partial_t A, \partial_t P, \partial_E$ remain small in the stable regime is a consistent assumption, thus making perturbative expansion valid. Any term above non-linear in $\partial_t A, \partial_t P, \partial_E$ is of higher order in this approximation. We wish to find the part of $\gamma AP/E\partial_t P$ that is linear in the first time derivative. In the stable regime changes are slow and thus a short time after the shock we can find the Taylor expansions for the variables near $t = 0$. Again, we rescale the variables at $t = 0$ to $E_0 = P_0 = A_0 = 1$. Using equation (8), we get:

$$\begin{aligned}\frac{A(t)P(t)}{E(t)}\partial_t P &= \frac{A_0P_0 + t(\partial_t A_0 P_0 + A_0 \partial_t P_0)}{E_0 + tA_0\partial_t P_0}\partial_t P \\ &\approx \frac{1}{E_0}\left(A_0P_0 + t(\partial_t A_0 P_0)\right)\partial_t P \\ &= \partial_t P + O(\partial_t A_0 \partial_t P) \approx \partial_t P \end{aligned} \tag{28}$$

Thus the assumption of smallness of the derivatives is consistent. We may use perturbation theory and safely discard the non-linear terms in finding the stability conditions. The stability condition is simply that ω^2 be positive.

We combine equations (6)-(8) in a 1-by-1 system by taking another ∂_t derivative from (7). Thus, we eliminate most occurrences of E and A an find an equation for P, which contains non-linear terms in it. Using the price change $u \equiv \partial_t P$ as the fundamental variable, the nonlinearities are roughly of type $u^2 + a\partial_t u^2$, described in the following equations:

$$\begin{aligned}&\left[\tau\partial_t^2 + \partial_t + \omega^2\right]u = \frac{O\left(u^2, \partial_t u^2\right)}{p} \\ &\frac{1}{\tau} = \frac{1}{\tau_A} + \frac{1}{\tau_P}, \quad \omega^2 = \frac{1 - \gamma\frac{AP}{E}}{\tau_A + \tau_P}.\end{aligned} \tag{29}$$

For a small shock $f_0 = -\varepsilon$ we may safely use $AP/E = 1$. Thus for a time-scale where $\gamma < 1$ and is not changing much we are essentially dealing with a damped harmonic oscillator. Notice that equation (29) is almost identical to what Bouchaud proposes in[30] to explain the 1987 crash.

Table S3. GIIPS debt data used in the analysis. All numbers are in million euros. Our data is based on two sources: 1) The EBA 2011 stress test data, which only includes exposure of European banks and funds (these are the ones where the "Code Name" is of the form CC123); 2) A list of top 50 global banks, insurance companies and funds with largest exposures to GIIPS debt by end of 2011 provided by S. Battiston et al. (These have a name as their "Code Name"), which was consolidated by the authors.

ID	Name	Code Name	Holdings	Equity	Greece	Italy	Portugal	Spain	Ireland
0	GESPASTOR	Gespastor	3.5e+02	1.6e+03	0	0	0	3.5e+02	0
1	M&G	M&G	37	1.1e+04	0	0	37	0	0
2	UNION INVESTMENT	Union Inv.	3.4e+03	7e+02	1.6e+02	2e+03	77	1e+03	1.5e+02
3	ATTICA BANK	Attica	1.8e+02	1.5e+04	1.8e+02	0	0	0	0
4	MILANO ASSICURAZIONI	Milano Assic.	74	9.3e+02	23	0	0.71	49	2.1
5	GROUPAMA	Groupama	4.4e+02	4.3e+03	0	4.2e+02	19	0	0
6	AEGON NV	Aegon	1.1e+03	2.6e+04	2	65	9	9.8e+02	26
7	RIVERSOURCE	River Source	48	7.4e+03	48	0	0	0	0
8	AVIVA PLC	Aviva	1.1e+04	1.8e+04	1.5e+02	8.4e+03	2.3e+02	1.4e+03	7.2e+02
9	EMPORIKI BANK	Emporiki	2.9e+02	1.2e+03	2.9e+02	0	0	0	0
10	MELLON GLOBAL	Mellon	16	2.8e+04	0	0	0	0	16
11	DAIWA	Daiwa	7.1e+02	7.3e+03	0	5e+02	0	2e+02	0
12	FIDEURAM	Fideuram	2e+03	5.5e+02	0	2e+03	0	0	0
13	UNIPOL	Unipol	1.3e+04	2.5e+03	26	1.2e+04	1.5e+02	1.1e+03	2.4e+02
14	WGZ BANK AG WESTDT. GENO.	DE029	3.6e+03	1.9e+03	3.2e+02	1.4e+03	4.6e+02	1.2e+03	2.2e+02
15	JYSKE BANK	DK009	1.2e+02	1.4e+04	64	0	19	15	22
16	OESTERREICHISCHE VOLKSBANK AG	AT003	3.7e+02	4.8e+02	1.1e+02	1.5e+02	29	66	13
17	CAIXA PORTUGAL	Caixa (PT)	8.1e+03	2.4e+04	35	4.6e+02	30	7.5e+03	44
18	BLACKROCK	Blackrock	2e+03	2e+04	1.2e+02	1.1e+03	29	7.1e+02	30
19	BANK OF AMERICA	BofA	3.8e+02	1.8e+05	13	2.5e+02	5.4	83	29
20	NORDEA BANK AB (PUBL)	SE084	1.6e+02	2.6e+04	0	97	0	64	1.4
21	CAJA DE AHORROS Y M.P.	ES077	1.5e+03	-	0	0	0	1.5e+03	0
22	SELLA GESTIONI	Sella	6.6e+02	1.3e+02	0	6.6e+02	0	0	0
23	MITSUBISHI UFJ	Mitsubishi	1.6e+03	8.1e+04	0	9.2e+02	71	5.2e+02	62
24	UBS	UBS	1.3e+03	4.8e+04	53	6.8e+02	55	4.4e+02	42
25	OPPENHEIMER	Oppenheimer	2.4e+02	3.8e+02	15	0	0	2.2e+02	0
26	VONTOBEL	Vontobel	18	1.2e+03	18	0	0	0	0
27	NOMURA	Nomura	39	1.8e+04	0	0	20	0	19
28	MACKENZIE	MacKenzie	15	3.4e+03	15	0	0	0	0
29	AGEAS	Ageas	5.3e+03	7.8e+03	6.4e+02	2e+03	1e+03	1.1e+03	5.1e+02
30	DEUTSCHE POSTBANK	De.Postbank	9.2e+02	5.7e+03	9.2e+02	0	0	0	0
31	MORGAN STANLEY	Morgan Sta.	4.6e+02	5e+04	0	4.6e+02	0	0	0
32	HELVETIA HOLDING	Helvetia	1e+03	3.6e+03	7.6	7.2e+02	18	2.4e+02	15
33	HWANG-DBS	Hwang	23	8.7e+02	23	0	0	0	0
34	ASSICURAZIONI GENERALI	Generali	1.7e+04	1.8e+04	1.3e+03	5.4e+03	3.1e+03	5.7e+03	1.7e+03
35	AMLIN PLC	Amlin	15	1.6e+03	0	0	0	15	0
36	SWISS LIFE HOLDING	Swiss Life	5.9e+02	7.5e+03	30	1.7e+02	77	1.8e+02	1.3e+02
37	PHOENIX GROUP	Phoenix	3.2e+02	2.8e+03	0	2.3e+02	11	76	2.2
38	PRICE T ROWE	PT Rowe	15	2.6e+03	0	0	0	0	15
39	AXA	Axa	2.9e+04	4.9e+04	7.6e+02	1.7e+04	1.5e+03	9.4e+03	7.5e+02
40	TOKIO MARINE	Tokio Marine	56	1e+04	0	0	30	0	26
41	ROTHSCHILD	Rothschild	1.1e+02	6e+02	61	0	52	0	0
42	TT ELTA AEDAK	TT Elta Aedak	27	9.3e+02	27	0	0	0	0
43	BALOISE	Baloise	7.9e+02	3.2e+03	84	2.7e+02	98	2.3e+02	1.1e+02
44	NATIXIS	Netaxis	3.3e+03	2.1e+04	4.3e+02	1.3e+03	3.9e+02	8.6e+02	3.9e+02
45	CREDIT AGRICOLE	FR014	1.7e+04	4.9e+04	6.6e+02	1.1e+04	1.2e+03	3.9e+03	1.6e+02
46	JULIUS BAER	Jul. Baer	1.2e+02	3.5e+03	68	0	0	0	57
47	FRANKLIN TEMPLETON	Franklin Temp.	5.1e+03	9.7e+03	0	0	0	0	5.1e+03
48	NOVA LJUBLJANSKA BANKA	SI057	1.7e+02	-	20	96	15	26	15
49	STATE STREET	State St.	51	1.6e+04	0	0	27	0	24
50	ALLIANZ	Allianz	3.8e+04	1e+05	6.2e+02	2.9e+04	7.5e+02	7.1e+03	4.9e+02
51	VIENNA INSURANCE	Vienna	93	5e+03	21	13	0	7	52
52	BANCO POPOLARE - S.C.	IT043	1.2e+04	33	87	1.2e+04	0	2e+02	0
53	COMMERZBANK AG	DE018	2e+04	2.5e+04	3.1e+03	1.2e+04	9.9e+02	4e+03	32
54	LEGAL & GENERAL	L&G	3.8e+02	6.3e+03	1.1	3.3e+02	6.6	35	4.4
55	EFFIBANK	ES063	3e+03	2.7e+03	37	0	16	2.9e+03	0

Continued on next page

ID	Name	Code Name	Holdings	Equity	Greece	Italy	Portugal	Spain	Ireland
56	INTESA SANPAOLO S.P.A	IT040	6.2e+04	6.4e+05	6.2e+02	6e+04	73	8.1e+02	1.1e+02
57	IRISH LIFE AND PERMANENT	IE039	1.9e+03	3.5e+03	0	0	0	0	1.9e+03
58	HSBC HOLDINGS PLC	GB089	1.5e+04	1.3e+05	1.3e+03	9.9e+03	1e+03	2e+03	2.9e+02
59	DANSKE BANK	DK008	1.2e+03	1.3e+05	1	5.8e+02	1.1e+02	1.2e+02	4.1e+02
60	ROYAL BANK OF SCOTLAND	GB088	1e+04	9.6e+04	1.2e+03	7e+03	2.9e+02	1.5e+03	4.5e+02
61	BNP PARIBAS	FR013	4.1e+04	8.6e+04	5.2e+03	2.8e+04	2.3e+03	5e+03	6.3e+02
62	BARCLAYS PLC	GB090	2e+04	8e+04	1.9e+02	9.4e+03	1.4e+03	8.8e+03	5.3e+02
63	LLOYDS BANKING GROUP PLC	GB091	94	5.8e+04	0	32	0	62	0
64	DEUTSCHE BANK AG	DE017	1.3e+04	5.5e+04	1.8e+03	7.7e+03	1.8e+02	2.6e+03	5.3e+02
65	SOCIETE GENERALE	FR016	1.8e+04	5.1e+04	2.8e+03	8.8e+03	9e+02	4.8e+03	9.8e+02
66	BPCE	FR015	8.5e+03	4.1e+04	1.3e+03	5.4e+03	3.5e+02	1e+03	3.4e+02
67	BBVA	ES060	6.1e+04	4e+04	1.3e+02	4.2e+03	6.6e+02	5.6e+04	0
68	BANK OF VALLETTA (BOV)	MT046	24	-	10	3.9	2.8	0	7
69	BANCO BPI, SA	PT056	5.5e+03	8.2e+02	3.2e+02	9.7e+02	3.9e+03	0	2.8e+02
70	BANCO SANTANDER S.A.	ES059	5.1e+04	2.6e+04	1.8e+02	7.2e+02	3.7e+03	4.6e+04	0
71	CAIXA DE AFORROS DE GALICIA,	ES067	4.7e+03	2.3e+04	0.0022	1.6e+02	1.3e+02	4.4e+03	0
72	CAIXA D’ESTALVIS DE CATALUNYA	ES066	2.8e+03	2.3e+04	0	0	0	2.8e+03	0
73	CAJA DE AHORROS Y PENSIONES	ES062	3.7e+04	2.2e+04	0	1.3e+03	26	3.5e+04	0
74	KBC BANK	BE005	7.9e+03	1.7e+04	4.4e+02	5.6e+03	1.6e+02	1.4e+03	2.7e+02
75	ERSTE BANK GROUP (EBG)	AT001	1.2e+03	1.5e+04	3.5e+02	6e+02	1e+02	1.4e+02	40
76	JP MORGAN	JPM	17	1.5e+05	0	0	17	0	0
77	BAYERISCHE LANDESBANK	DE021	1.3e+03	1.4e+04	1.5e+02	5.1e+02	1.1e-05	6.6e+02	20
78	BFA-BANKIA	ES061	2.5e+04	1.2e+04	55	0	0	2.5e+04	0
79	SNS BANK NV	NL050	1e+03	5.4e+03	47	7.6e+02	0	57	1.6e+02
80	RAIFFEISEN BANK (RBI)	AT002	4.6e+02	1.1e+04	1.7	4.5e+02	2.1	3.5	0.00016
81	DZ BANK AG DT.	DE020	8.7e+03	1.1e+04	7.3e+02	2.7e+03	1e+03	4.2e+03	51
82	F VAN LANSCHOT	Lanschot	18	7.4e+02	0	0	0	0	18
83	ALLIED IRISH BANKS PLC	IE037	6.5e+03	1.4e+04	40	8.2e+02	2.4e+02	3.3e+02	5e+03
84	SKANDINAVISKA ENSKILDA BANKEN	SE085	6.3e+02	1.2e+04	1.2e+02	2.9e+02	1.3e+02	86	0
85	IBERCAJA	Ibercaja	9.6e+02	2.7e+03	0	0	0	9.6e+02	0
86	LANDESBANK BADEN-WURT...	DE019	2.8e+03	9.5e+03	7.8e+02	1.4e+03	95	5.4e+02	0
87	BANCO POPULAR ESPANOL, S.A.	ES064	9.7e+03	9.1e+03	0	2.1e+02	6.4e+02	8.9e+03	0
88	CAJA ESP. DE INVER. SALAMANCA	ES070	7.6e+03	-	0	0	27	7.6e+03	0
89	NORDDEUTSCHE LANDESBANK	DE022	2.8e+03	6.5e+03	1.5e+02	1.9e+03	2.6e+02	5e+02	41
90	BANCA MARCH, S.A.	ES079	1.5e+02	6.5e+03	0	0	0	1.5e+02	0
91	OP-POHJOLA GROUP	FI012	43	6.2e+03	3.1	0.36	0.00093	0.07	40
92	BANCO COMERCIAL PORTUGUES,	PT054	7.4e+03	4.4e+03	7.3e+02	50	6.5e+03	0	2.1e+02
93	BANCO DE SABADELL, S.A.	ES065	7.4e+03	5.9e+03	0	0	91	7.3e+03	38
94	HYPO REAL ESTATE HOLDING AG,	DE023	1.1e+04	-	0	7.1e+03	4.9e+02	3.4e+03	44
95	FRANKLIN ADVISERS INC	Franklin Adv.	3.6e+02	4.7e+02	0	0	0	0	3.6e+02
96	ABN AMRO BANK NV	NL049	1.5e+03	2.8e+02	0	1.3e+03	0	1.1e+02	1.3e+02
97	MUENCHENER RV	Munich RV	8.2e+03	2.3e+04	5.8e+02	3.6e+03	4.2e+02	1.9e+03	1.8e+03
98	HSH NORDBANK AG, HAMBURG	DE025	1e+03	4.8e+03	1e+02	6.6e+02	62	1.8e+02	0
99	GRUPO BANCA CIVICA	ES071	4.8e+03	-	5.4	0	0	4.7e+03	0
100	CAIXA GERAL DE DEPOSITOS, SA	PT053	6.8e+03	5.3e+03	51	0	6.5e+03	2e+02	23
101	CAJA DE AHORROS DEL MEDITER...	ES083	5.6e+03	3.8e+03	0	20	4.8	5.6e+03	15
102	GRUPO BMN	ES068	3.7e+03	-	0	0	88	3.6e+03	0
103	BANK OF IRELAND	IE038	5.6e+03	1e+04	0	30	0	0	5.6e+03
104	DEKABANK	DE028	6e+02	3.3e+03	87	2.7e+02	32	1.8e+02	30
105	DEXIA	BE004	2.3e+04	3.3e+03	3.5e+03	1.6e+04	1.9e+03	1.5e+03	0.34
106	GRUPO BBK	ES075	3.1e+03	-	0	0	3	3.1e+03	4
107	BANKINTER, S.A.	ES069	3.6e+03	3.1e+03	0	1.2	0	3.6e+03	0
108	WESTLB AG, DUSSELDORF	DE024	2.2e+03	3e+03	3.4e+02	1.1e+03	0	7.5e+02	35
109	UNIONE DI BANCHE ITALIANE SCPA	IT044	1.1e+04	1.1e+04	25	1.1e+04	0	0	0
110	CAJA DE AHORROS Y M.P.	ES072	3.3e+03	2.7e+03	0	3.8e+02	0	2.9e+03	0
111	CAIXA D’ESTALVIS UNIO DE CAIXES	ES076	2.6e+03	-	0	11	0	2.6e+03	13
112	BANK OF CYPRUS PUBLIC CO	CY007	2.8e+03	2.4e+03	2.4e+03	36	0	58	3.2e+02
113	LANDESBANK BERLIN AG	DE027	1.1e+03	2.3e+03	4.5e+02	3.3e+02	0	3.7e+02	0.075
114	ALPHA BANK	GR032	5.5e+03	2e+03	5.5e+03	0	0	0	0
115	UNICREDIT S.P.A	IT041	5.2e+04	9.3e+05	6.7e+02	4.9e+04	94	1.9e+03	58
116	MARFIN POPULAR BANK PUBLIC CO	CY006	3.4e+03	1.7e+03	3.4e+03	0	0	0	39
117	BANCO PASTOR, S.A.	ES074	2.6e+03	1.6e+03	41	1e+02	1.2e+02	2.3e+03	0
118	GRUPO CAJA3	ES078	1.5e+03	-	0	0	0	1.5e+03	8.4
119	TT HELLENIC POSTBANK S.A.	GR035	5.3e+03	9.3e+02	5.3e+03	0	0	0	0
120	EFG EUROBANK ERGASIAS S.A.	GR030	8.9e+03	8.8e+02	8.8e+03	1e+02	0	0	0
121	ESPIRITO SANTO GROUP,	PT055	3.1e+03	6.2e+03	3.1e+02	0	2.7e+03	55	0

Continued on next page

ID	Name	Code Name	Holdings	Equity	Greece	Italy	Portugal	Spain	Ireland
122	AGRICULTURAL BANK OF GREECE	GR034	7.9e+03	7.5e+02	7.9e+03	0	0	0	0
123	CAJA DE AHORROS DE VITORIA	ES080	6e+02	-	0	0	0	6e+02	0
124	ING BANK NV	NL047	1.1e+04	3.5e+04	7.5e+02	7.7e+03	7.6e+02	1.9e+03	92
125	RABOBANK NEDERLAND	NL048	1.1e+03	-	3.8e+02	4.4e+02	82	1.6e+02	60
126	WUERTTEMBERGISCHE LV	Wuetter. LV	7.7e+02	1.2e+02	85	4.5e+02	52	1.8e+02	8
127	NYKREDIT	DK011	1.1e+02	-	22	88	0	0	0
128	MONTE DE PIEDAD Y CAJA	ES073	3.3e+03	58	6	3.1e+02	0	2.9e+03	0
129	CAJA DE AHORROS Y M.P.	ES081	6	58	0	0	0	6	0
130	BANCA MONTE DEI PASCHI DI	IT042	3.3e+04	1.9e+03	8.1	3.2e+04	2e+02	2.8e+02	0
131	COLONYA - CAIXA D'ESTALVIS DE	ES082	26	-	0	0	0	26	0
132	BANQUE ET CAISSE D'EPARGNE DE	LU045	2.8e+03	2.9e+03	85	2.4e+03	1.8e+02	1.7e+02	0
133	PIRAEUS BANK GROUP	GR033	8.2e+03	-1.9e+03	8.2e+03	0	0	0	0
134	NATIONAL BANK OF GREECE	GR031	1.9e+04	-4.3e+03	1.9e+04	0	0	0	18
135	ZURICH FINANCIAL	Zurich	8.7e+03	2.5e+04	0	4.2e+03	3.7e+02	3.7e+03	3.7e+02
136	MITSUI	Mitsui	6.4e+02	6.9e+04	0	3.7e+02	25	1.7e+02	76
